# Enhancement of Seawater Stress Tolerance in Barley by the Endophytic Fungus *Aspergillus ochraceus*

**DOI:** 10.3390/metabo11070428

**Published:** 2021-06-29

**Authors:** Ali A. Badawy, Modhi O. Alotaibi, Amer M. Abdelaziz, Mahmoud S. Osman, Ahmed M. A. Khalil, Ahmed M. Saleh, Afrah E. Mohammed, Amr H. Hashem

**Affiliations:** 1 Botany and Microbiology Department, Faculty of Science, Al-Azhar University, Nasr City, Cairo 11884, Egypt; ali.abdalhalim@azhar.edu.eg (A.A.B.); amermorsy@azhar.edu.eg (A.M.A.); ahmed_khalil@azhar.edu.eg (A.M.A.K.); 2 Department of Biology, College of Science, Princess Nourah bint Abdulrahman University, Riyadh 84428, Saudi Arabia; afamohammed@pnu.edu.sa; 3 Biology Department, College of Science, Taibah University, Yanbu 41911, Saudi Arabia; 4 Department of Botany and Microbiology, Faculty of Science, Cairo University, Giza 12613, Egypt; asaleh@sci.cu.edu.eg

**Keywords:** endophytes, barley plants, salinity, fungal endophytes, *Aspergillus ochraceus*, plant growth regulation

## Abstract

Symbiotic plant-fungi interaction is a promising approach to alleviate salt stress in plants. Moreover, endophytic fungi are well known to promote the growth of various crop plants. Herein, seven fungal endophytes were screened for salt tolerance; the results revealed that *Aspergillus ochraceus* showed a great potentiality in terms of salt tolerance, up to 200 g L^−1^. The indole acetic acid (IAA) production antioxidant capacity and antifungal activity of *A. ochraceus* were evaluated, in vitro, under two levels of seawater stress, 15 and 30% (*v*/*v*; seawater/distilled water). The results illustrated that *A. ochraceus* could produce about 146 and 176 µg mL^−1^ IAA in 15 and 30% seawater, respectively. The yield of IAA by *A. ochraceus* at 30% seawater was significantly higher at all tryptophan concentrations, as compared with that at 15% seawater. Moreover, the antioxidant activity of ethyl acetate extract of *A. ochraceus* (1000 µg mL^−1^) at 15 and 30% seawater was 95.83 ± 1.25 and 98.33 ± 0.57%, respectively. Crude extracts of *A. ochraceus* obtained at 15 and 30% seawater exhibited significant antifungal activity against *F. oxysporum,* compared to distilled water. The irrigation of barley plants with seawater (15 and 30%) caused notable declines in most morphological indices, pigments, sugars, proteins, and yield characteristics, while increasing the contents of proline, malondialdehyde, and hydrogen peroxide and the activities of antioxidant enzymes. On the other hand, the application of *A. ochraceus* mitigated the harmful effects of seawater on the growth and physiology of barley plants. Therefore, this study suggests that the endophytic fungus *A. ochraceus* MT089958 could be applied as a strategy for mitigating the stress imposed by seawater irrigation in barley plants and, therefore, improving crop growth and productivity.

## 1. Introduction

Crop plants are often exposed to unfavorable environmental conditions, including abiotic stresses that lead to annual losses in crop productivity all over the world [1,2]. Salinity is one of the major abiotic stresses that occur in irrigated and non-irrigated regions, leading to fierce effects on plant growth and production, particularly in crop plants which exhibited a reduction in seed germination, plant growth, and plant biomass [3]. The continued exposure of plants to salt stress resulted in the toxicity of specific ions and nutrients, hormonal imbalance, and a decrease in water potential [4]. Salinity is a growing problem in several countries around the world [5,6,7]. Plants grown under salinity stress conditions accumulate excessive amounts of salt, generating physicochemical disturbances resulting in oxidative stress, suppression of photosynthesis, generation of reactive oxygen species (ROS), and metabolic disorders, all of which contribute to a decrease in plant growth and yields [8,9].

The enhancement of plant productivity by abiotic stress resistance strategies is a great approach to sustainable agriculture because it reduces the chemical input [10]. Endophytic fungi are part of this strategy, enhancing crop yield and quality by reducing the abiotic stress. Fungal endophytes are eukaryotic organisms that exist in living healthy plant tissues at some stages of their lifecycle, without producing any apparent symptoms or obvious harm effects to their hosts [11]. Fungal endophytes obtain their food and protection from the plant, while the plant increases its resistance against the abiotic and biotic stresses through this symbiotic interaction [12]. Fungal endophytes play an important role in plant growth promotion by (1) solubilizing some macronutrients, as potassium, phosphorus and zinc, (2) atmospheric nitrogen fixing, and (3) the production of siderophores, phytohormones (auxins, gibberellins, etc.), hydrogen cyanide, antioxidant compounds, and ammonia [13]. Moreover, fungal endophytes play a major role as biocontrol agents toward phytopathogenic fungi such as *Fusarium oxysporum* [14], *F. solani* [15], *Verticillium dahlia* [16], and *Rhizoctonia solani* [17]. Many endophytic fungi were used for plant growth promotion as well as biocontrol agents—for instance, *Chaetomium globosum* and *Trichoderma harzanium* [18], *Aspergillus parasiticus* [19], *Aspergillus ustus* [20], *Aspergillus terreus JF27* [21], and *Aspergillus niger* [22]. Fungal endophytes enhance plant vigor by resisting herbivory and promote plant growth by increasing the nutrient uptake and water use efficacy and by decreasing environmental stresses [23]. The application of an endophytic fungus, *Aspergillus flavus* CHS1, significantly increased the chlorophyll content, root-shoot length, and biomass production in soybean plants under NaCl stress, up to 400 mM, by modulating the levels of endogenous plant hormones and the activities of antioxidant enzymes. [10].

Barley (*Hordeum vulgare*) is a member of the Poaceae family (Gramineae). It is the fourth major cereal crop after wheat, corn, and rice in terms of production. It is widely used to feed humans and animals, as well as in the production of malt [24,25]. The importance of using barley as a food is mainly due to its health benefits, in addition to being an excellent source of dietary fiber and a functional food ingredient. Barley is cultivated in around 100 different countries [26,27].

This study aimed to use *Aspergillus ochraceus* MT089958 as an endophytic fungus for the first time to regulate the growth of barley, especially under seawater irrigation conditions, in addition to its ability to increase the barley plant’s tolerance and resistance to salt stress conditions.

## 2. Results

### 2.1. Screening of Salt-Tolerant Endophytic Fungi

The results in Table 1 show that *Aspergillus ochraceus* was found to be the most salt-tolerant fungus, capable of tolerating up to 200 g L^−1^ of sodium chloride up to. Moreover, *Alternaria tenuissima* and *Curvularia lunata* could grow up to 150 g L^−1^ in the presence of sodium chloride. On the other hand, *Aspergillus hiratsukae*, *Chaetomium* sp., and *Chaetomium globosum* ranked lowest among the other fungal strains that tolerate sodium chloride. From these results, *A. ochraceus* MT089958 was selected for further experiments in vitro and in vivo due to its ability to tolerate high concentrations of salt compared with other endophytic fungal strains.

### 2.2. Indole Acetic Acid (IAA) Production

**Figure 1:**
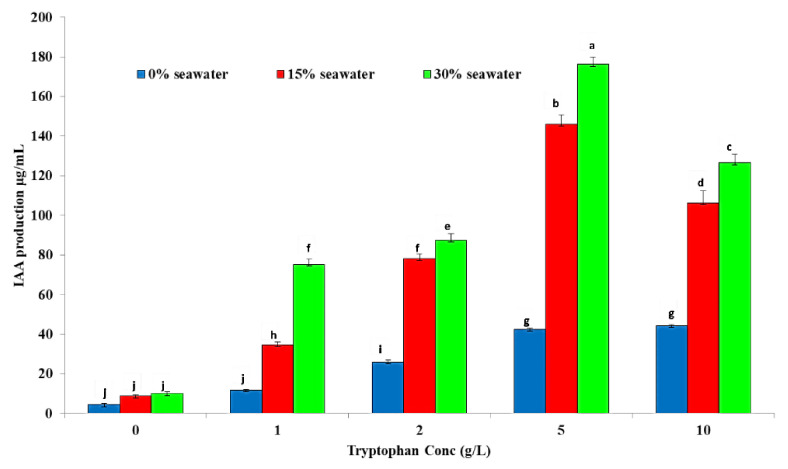
IAA production by *A. ochraceus* at different levels of salinity. Different letters indicate significant differences between the treatments according to an LSD test at *p* < 0.05.

The indole acetic acid production from *A. ochraceus* in 0, 15, and 30% seawater in the presence and absence of tryptophan is shown in Figure 1. Results revealed that *A. ochraceus* has the ability to produce IAA in the presence and absence of tryptophan, while IAA was higher in the presence of tryptophan rather than in its absence. In the case of 0% seawater, IAA increased with the increase of tryptophan concentration, reaching 44 µg mL^−1^ at a tryptophan concentration of 10 g L^−1^. On the other hand, when *A. ochraceus* is grown on a medium containing 15 and 30% seawater separately, it leads to a significant increase in IAA production compared to 0% seawater. The yield of IAA by *A. ochraceus* at 30% seawater was significantly higher than that at 15% seawater at all tryptophan concentrations, as shown in Figure 1. In 15% seawater the yield of IAA at 0, 1, 2, 5, and 10 g L^−1^ tryptophan was 8.8, 34.7, 78.3, 246, and 154 µg mL^−1^, while at 30% seawater the yield was 10, 75.3, 87.6, 276, and 226 µg mL^−1^, respectively. Previous data showed that 30% seawater was the best for IAA production by *A. ochraceus* at 5 g L^−1^ tryptophan.

### 2.3. Antioxidant Production

**Figure 2:**
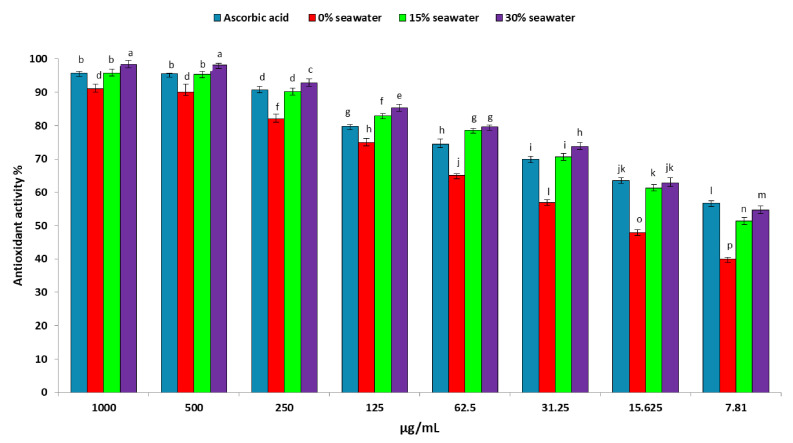
Antioxidant activity of the crude extract of *A. ochraceus* cultured on different levels of salinity. Different letters indicate significant differences between the treatments according to an LSD test at *p* < 0.05.

In this study, the antioxidant potential of *A. ochraceus* on 0, 15, and 30% seawater was evaluated using the DPPH method as shown in Figure 2. The results showed that when *A. ochraceus* was grown on a medium containing 0% seawater (without salt stress). It exhibited significant antioxidant activities compared to the standard (ascorbic acid) and tended to increase its activities with increasing concentration. Moreover, in the presence of salt stress (15 and 30% seawater), *A. ochraceus* exhibited an antioxidant activity that was significantly higher than the one listed in the absence of salt stress. Results showed that the antioxidant activity of an ethyl acetate extract of *A. ochraceus* (1000 µg mL^−1^) at 15 and 30% seawater was 95.83 ± 1.25 and 98.33 ± 0.57%, while the lowest concentration (7.81 µg/mL) exhibited an antioxidant activity of 51.33 and 54.7, respectively. These results indicate that the fungal extract of *A. ochraceus* exhibited a strong antioxidant activity at high and low concentrations under salt stress conditions (15 and 30% seawater).

### 2.4. Antifungal Activity

**Figure 3:**
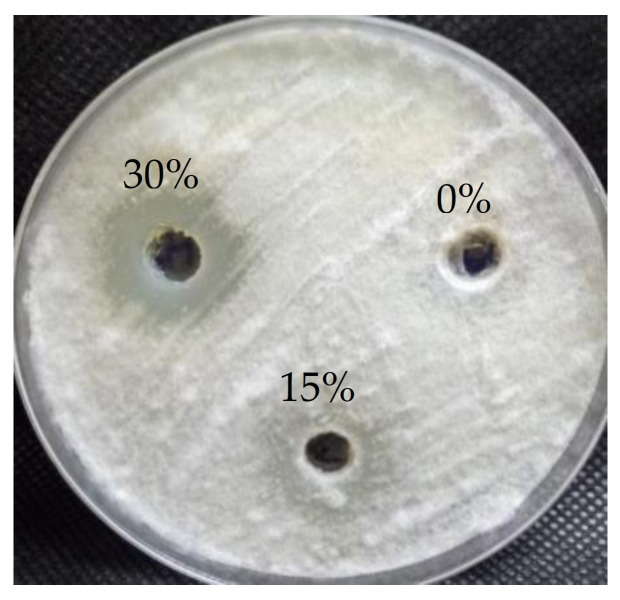
Inhibition zones caused by the crude extracts of *A. ochraceus* cultured at different levels of salinity (0, 15, and 30% seawater) against *F. oxysporum*.

In the current study, the antifungal activity of *A. ochraceus* at different concentrations of seawater (0, 15, and 30%) was assessed against *F. oxysporun* by the agar well diffusion assay method, as shown in Figure 3 and Table 2 Results showed that salinity at different levels significantly affects antifungal activity, and that a salinity increase leads to an increase in the antifungal activity of *A. ochraceus*, and vice versa. Moreover, the results show that *A. ochraceus* at 0% seawater did not exhibit any antifungal activity on *F. oxysporum*. On the other hand, *A. ochraceus* at 15 and 30% seawater exhibited antifungal activity. The activity at 30% was significantly higher than that at 15% seawater, and the inhibition zones at 15 and 30% seawater were 17.3 ± 0.57 and 23.6 ± 1.15 mm. Furthermore, MIC was determined at 15 and 30% seawater. It was 250 µg mL^−1^ at 15% and 62.5 µg mL^−1^ at 30%. *A. ochraceus* exhibited a potential antifungal activity under salinity stress due to being isolated from the halotolerant mangrove plant.

### 2.5. Aspergillus Ochraceus Application Promoted the Growth of Barley Plants under Salinity Stress Conditions

**Figure 4:**
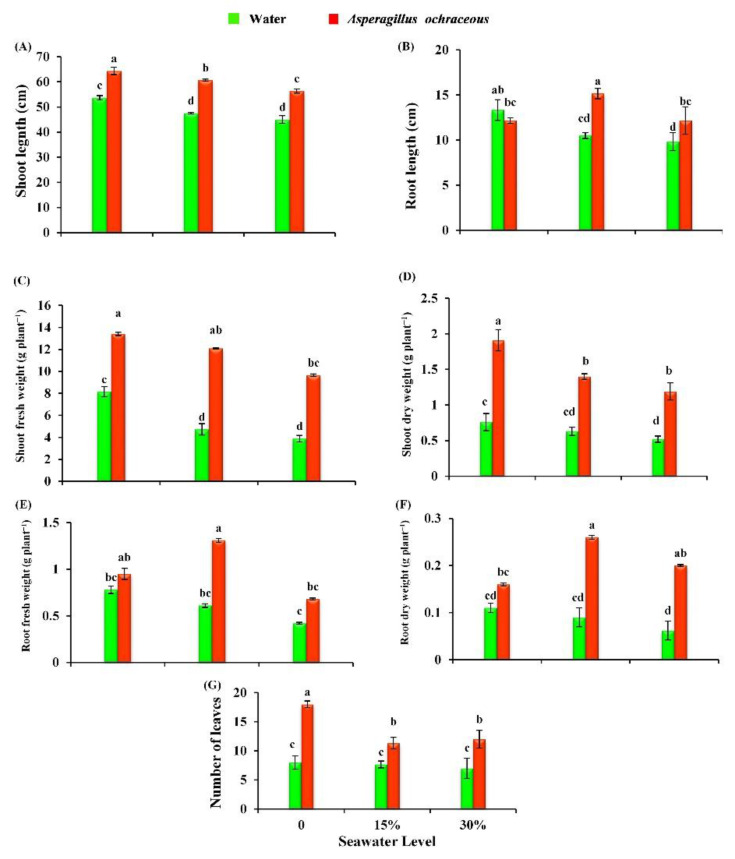
Effect of seawater irrigation, *A. ochraceus* application, and their interactions on the morphological parameters of barley plants {(**A**) shoot length, (**B**) root length, (**C**) shoot fresh weight, (**D**) shoot fresh weight, (**E**) root fresh weight, (**F**) root dry weight, and (**G**) number of leaves}. Each bar represents the mean ± standard error. Different letters indicate significant differences between the treatments according to an LSD test at *p* < 0.05.

The changes in the morphological parameters of barley plants in response to the irrigation with seawater (15% and 30%) and the application of *Aspergillus ochraceus* are clarified in Figure 4. The irrigation with 15 and 30% seawater significantly decreased the shoot length (by 11.48 and 16.14%), root length (by 21.24 and 26.26%), shoot fresh weight (by 42.09 and 52.4%), shoot dry weight (by 17.02 and 31.57%), root fresh weight (by 21.79 and 46.15%), root dry weight (by 18.18 and 43.63%), and number of leaves (by 4.25 and 12.5%), respectively, of barley plants as compared to control plants (0% seawater). The obtained results, in Figure 4, also clarified that the application of the *A. ochraceus* fungus increased the morphological characters of non-stressed wheat plants (control), and the most significant increases were found in shoot length (by 19.88%), shoot fresh weight (by 64.54%), shoot dry weight (by 151.31%), and number of leaves (by 45.45%). Regarding the interaction between salinity levels and *A. ochraceus* treatment, the fungus treatment promoted all the tested parameters of barley morphology compared to salinity-stressed plants.

### 2.6. Aspergillus Ochraceus Application Enhanced Leaf Pigments of Barley Plants under Salinity Stress Conditions

**Figure 5:**
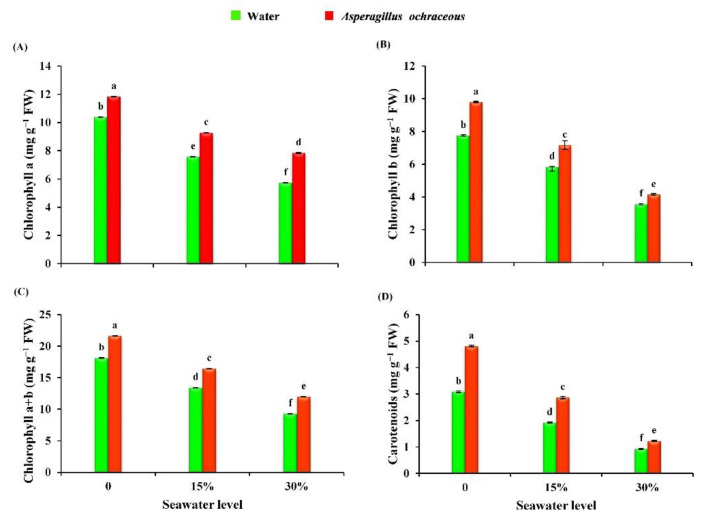
Effect of seawater irrigation, *A. ochraceus* application, and their interactions on leaf pigments of barley plants {(**A**) chlorophyll a, (**B**) chlorophyll b, (**C**) chlorophyll a + b, and (**D**) carotenoids}. Each bar represents the mean ± standard error. Different letters indicate significant differences between the treatments according to an LSD test at *p* < 0.05. FW: fresh weight.

The influence of seawater (15% and 30%) and *A. ochraceus* on photosynthetic pigments of barley plants is illustrated in Figure 5. The irrigation with seawater at 15% and 30% concentrations exhibited a significant decrease in the contents of chlorophyll a (by 26.98 and 44.89%), chlorophyll b (by 24.64 and 54.06%), total chlorophylls (by 25.92 and 48.75%), and carotenoids (by 37.86 and 69.90%) in barley plants as compared with unstressed plants (0% seawater). It has been observed that the irrigation with seawater at 30% recorded the lowest decrease in the measured parameters. On the other hand, applying endophytic *A. ochraceus* as treatment caused significant increases in the contents of photosynthetic pigments of non-stressed barley plants, compared to controls (see Figure 5). Furthermore, salinity-stressed barley plants (15, 30% seawater irrigated plants) observed significant enhancements in their contents ofchlorophyll a (by 22.42 and 37.06%), chlorophyll b (by 22.77 and 16.57%), total chlorophyll (by 22.56 and 29.17%), and carotenoids (by 49.47 and 32.25%) as a result of the *A. ochraceus* endophytic fungus treatment compared to untreated plants.

### 2.7. Aspergillus Ochraceus Application Affected the Soluble Sugar, Protein, and Proline Contents of Barley Plants under Salinity Stress Conditions

#### 2.7.1. Soluble Sugars

The impact of seawater (15% and 30%) and the endophytic fungus *Aspergillus ochraceus* on the contents of soluble sugars in barley are shown in Table 3. There is a significant reduction in soluble sugar contents in barley plants because of the irrigation with seawater either at 15% or 30% concentrations (by 15.44 and 24.11), respectively. Regarding the interaction between seawater irrigation and the fungus treatment, the application of the endophytic fungus *A. ochraceus* was found to be able to increase the contents of sugars in seawater-stressed barley plants (by 10.27 and 14.27%) compared with un-treated plants.

#### 2.7.2. Soluble Proteins

The obtained results, in Table 3, illustrate the effect of seawater (15% and 30%) and the endophytic fungus *A. ochraceus* on soluble protein contents in barley plants. It was clarified that barley plants which were irrigated with seawater at 15% and 30% concentrations exhibited a significantly lower content of soluble protein (by 31.68 and 43.15%) when compared to unstressed plants (control). Contrarily, the application of the plant-growth-promoting fungus *A. ochraceus* individually or under salinity stress conditions led to improvements in the soluble protein contents of barley plants.

#### 2.7.3. Free Proline

The impact of seawater stress (15% and 30%), the endophytic fungus *Aspergillus ochraceus*, and their interactions on the contents of free proline in barley plants are clarified in Table 3. The amino acid proline, that acts as an osmo-protectant, has been increased in plants subjected to salinity stress. Results illustrate that proline contents were significantly increased in response to the irrigation of barley plants with seawater at both concentrations (15% and 30%), by 131.57 and 163.15%. On the other hand, the utilization of the endophytic fungus *A. ochraceus* under seawater stress at either 15% or 30% concentrations decreased the amount of proline in barley plants (by 37.5 and 41%) compared to plants stressed by salinity and untreated with the endophyte fungus (Table 4). Likewise, proline was markedly decreased in *Aspergillus aculeatus*-inoculated barley plant leaves in comparison with non-inoculated plants.

### 2.8. Aspergillus Ochraceus Application Boosted the Activity of the Antioxidant Enzymes of Barley Plants under Salinity Stress Conditions

**Figure 6:**
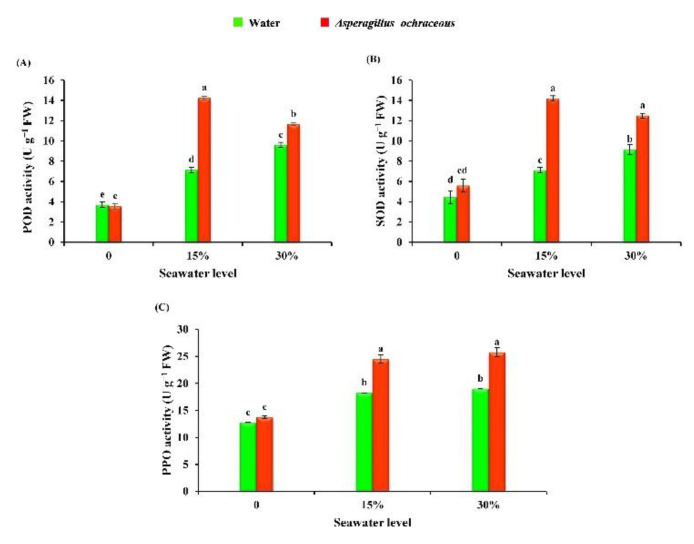
Effect of seawater irrigation, *A. ochraceus* application, and their interactions on the activity of antioxidant enzymes of barley plants {(**A**) POD, (**B**) SOD, and (**C**) PPO}. Each bar represents the mean ± standard error. Different letters indicate significant differences between the treatments according to an LSD test at *p* < 0.05. FW: Fresh weight.

The statistical analysis of our data showed the significant impact of seawater (15% and 30%) irrigation and the endophytic fungus (*Aspergillus ochraceus*) treatment on the activities of the antioxidant enzymes of barley plants (Figure 6). It was observed that the activities of peroxidase (POD), superoxide dismutase (SOD), and polyphenol oxidase (PPO) were significantly enhanced due to the irrigation of barley plants with seawater at either 15% (by 93.24, 59.55 and 43.13%), respectively, or 30% (by 159.45, 105.61 and 49.01%), in comparison with the unirrigated plants. Obviously, a seawater concentration of 30% stimulates the activities of the mentioned antioxidant enzymes more than the 15% seawater concentration. Regarding the treatment with *A. ochraceus*, the results in Figure 6 show that there are no significant changes in the activities of antioxidant enzymes when barley plants are individually treated with *A. ochraceus* in comparison with controls (un-treated plants) in normal conditions. Under seawater stress conditions, the endophytic fungus *A. ochraceus* exhibits a significant boosting in the activities of antioxidant enzymes (POD, SOD, and PPO) in barley plants either at 15% (by 99.3, 100 and 34.24%) or 30% seawater concentrations (by 21.35, 36.61 and 35.52%).

### 2.9. Aspergillus Ochraceus Application Suppressed the Malondialdehyde and Hydrogen Peroxide Contents of Barley Plants under Salinity Stress Conditions

**Figure 7:**
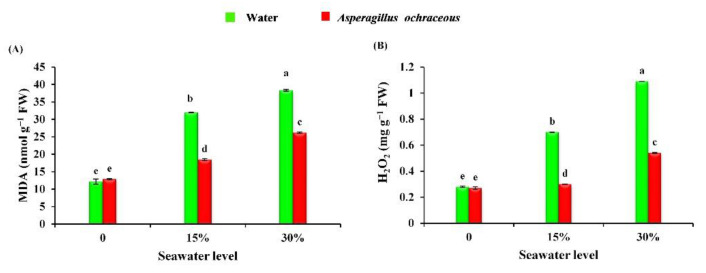
Effect of seawater irrigation, *A. ochraceus* application, and their interactions on the contents of (**A**) malondialdehyde (nmol g^−1^ FW) and (**B**) hydrogen peroxide (mg g^−1^ FW) in barley plants. Each bar represents the mean and standard error. The letters indicate significant differences between the treatments according to an LSD test at *p* < 0.05. FW: fresh weight.

Under salinity stress conditions, the observed results, in Figure 7, showed that salinity stress contributed to a strong increase in the contents of malondialdehyde (MDA) (by 162.94 and 214.95%) and hydrogen peroxide (H_2_O_2_) (by 150 and 289.28%) at two salinity levels (15, 30% seawater), when compared to control plants. However, the application of *Aspergillus ochraceus* significantly decreased the contents of malondialdehyde (by 42.28 and 31.72%) and hydrogen peroxide (by 57.14 and 50.45%) in salinity-stressed barley plants when compared to untreated salinity-stressed plants.

### 2.10. Aspergillus Ochraceus Application Increased the Yield Attributes of Barley Plants under Salinity Stress Conditions

Salinity stress significantly decreased the spike length (by 20.28 and 37.48%), spike weight (by 38.31 and 54.92%), number of grains (by 41.2 and 67.07%), grains weight (by 38.54 and 50%), and 1000 grains weight (by 22.74 and 53.19%) Table 4. Seed priming with *A. ochraceus* ameliorated the deleterious effects of salinity stress (15%, 30% seawater) on spike length (by 19.37 and 25.6%), spike weight (by 58.2 and 33.94%), number of grains (by 20.59 and 31.08%), grains weight (by 38.54 and 50%), and 1000 grains weight (by 22.74 and 53.19%). Regarding the application of *A. ochraceus*, the results in Table 4 show that there is a significant increase in the yield parameters when barley plants are individually treated with *A. ochraceus*, in comparison with controls (un-treated plants) in normal conditions.

## 3. Discussion

The evaluation of the tolerance of fungal strains to the saline environment has received great attention in recent years [28,29]. Moreover, the tolerance of fungal endophytes to different hyper-saline concentrations of sodium chloride was reported by several recent studies [28,30,31,32,33]. In the current study, *A. ochraceus* could tolerate sodium chloride up to 200 g L^−1^, and the tolerance to saline stress is explained by different mechanisms. Firstly, by the influence exerted on its symbiotic partners to adapt to a similar range of environments [33]. Secondly, because most fungi demand passive mechanisms such as the production of extracellular polysaccharides to cover the cells or increase the thickness of the cell wall, and the creation of cell clumps to survive at high salt concentrations [34]. Thirdly, through the production of IAA reported by several recent studies, which mention the effect of salt and heavy metal stress on the increasing IAA production by fungi in vitro [35,36,37]. Our findings are in accordance with Ahmad et al. [38], who reported that tryptophan is considered as a precursor of IAA biosynthesis and that its addition to the culture medium enhances IAA production. Furthermore, Turbat et al. [39] isolated fungal endophytes that have the ability to produce IAA in the presence or absence of tryptophan. These findings are in full agreement with Fouda et al. [40], who found an increase in IAA production by *Penicillium chrysogenum* and *Alternaria alternate*, though increasing the tryptophan concentration from 1 to 5 mg mL^−1^. Fourthly, endophytic fungi have the ability to produce antioxidant compounds inside plants, which can reduce the effect of ROS and help protect plants against biotic and abiotic stresses [41]. Biological reactions usually produce ROS as a by-product, causing cell death due to oxidative damage to biological materials [42]. In this context, similar findings have been reported by many studies that illustrated the antioxidant properties of endophytic fungi, particularly in mangrove plants [43,44,45]. The increasing antioxidant activity of *A. ochraceus* under salt stress is due to the stress that induces the fungus to activate the antioxidant system by producing antioxidant compounds that prevent the damage of ROS [46,47]. Fifthly, fungal endophytes produce different bioactive metabolites, which protect their host plants from microbial infections [48]. Furthermore, Rashmi et al. [49] reported that mangrove fungi can produce different bioactive compounds which can help plants cope with extreme environmental conditions.

Salinity stress affects the growth and metabolism of plants and causes a reduction in most growth indices. The decline in the growth parameters as a result of salinity stress was recorded in different investigations [50,51,52]. Our findings are in accordance with those documented by Zhang et al. [53], who found that plant height, root length, shoot fresh and dry weight, and root fresh and dry weight were markedly decreased as a result of salt stress. Recently, the experimental study of Osman et al. [6] demonstrated a reduction in the morphological related-indices of saline-stressed soybean plants. Chung et al. [54] explained that the reduction in the morphological parameters may be due to the ion toxicity and osmotic stress resulting from salinity exposure. With respect to the application of endophytic fungi, a study by Desai et al. [55] reported increases in the lengths of the roots and shoots of wheat and chickpea plants in response to the application of *Aspergillus* sp. Moreover, Asaf et al. [10] documented that the plant length and the fresh and dry weight of soybean plants were significantly increased due to the application of the endophytic fungus *Aspergillus flavus* CHS1 either under salinity stress or normal conditions. Other studies also reported the utilization of endophytic fungi in enhancing plant tolerance to salinity stress [56,57,58,59]. Such promotional effect of *A. ochraceus* upon the growth parameters might be due to its ability to produce phytohormones such as indole acetic acid, which not only regulate plant growth but also play a vital role in promoting plant growth under normal or unfavorable conditions, including salinity.

Pigments like chlorophylls and carotenoids were decreased in salinity-stressed barley plants. On this issue, a study of Zhang et al. [53] demonstrated that the contents of leaf pigments (chlorophyll a, b, total chlorophyll, and carotenoids) of wheat plants were lower than those of controls due to saline stress exposure. Similarly, the study of Jan et al. [52] evidenced that salinity stress significantly decreased the contents of chlorophylls and carotenoids of maize plants. Additionally, previous studies demonstrated that the content of plant photosynthetic pigments generally decreases under different salinity conditions [6,60,61]. The decline of leaf pigments may be related to the oxidation of chloroplast pigments, instability in the pigment-protein complex [62], or the activity of degrading enzymes under salinity stress [63]. Regarding the application of the endophytic fungus *A. ochraceus*, our results are in accordance with the results in [50,58,64]. The experimental study of Vafadar et al. [65] documented that the application of plant-growth-promoting fungi enhanced the contents of chlorophylls in candy leaf (*Stevia rebaudiana*) plants. The contents of chlorophyll a, b, and total chlorophyll in wheat plants stressed with NaCl were not only promoted but also reached a content similar to that of the control, after being treated with the plant-growth-promoting fungus *Trichoderma longibrachiatum* T6 [53]. Additionally, the inoculation of maize plants with the endophytic fungus *Meyerozyma caribbica* mitigated salinity stress [52]. The mitigation role of endophytic fungi may be attributed to the fact that they promote the biosynthesis of phytohormones and chlorophyll enzymes under different stressful conditions [66,67,68].

The synthesis of compatible osmolytes is among the different reactions of plants to salt stress. This allows cells to minimize the oxidative damage caused by ROS in response to high salinity [6,9]. Previous studies reported a significant reduction in the soluble sugar content due to salinity stress [6,51,69,70]. In plants exposed to salinity, soluble sugar contents were markedly diminished in comparison with plants not exposed to salinity [69]. A study by Goicoechea et al. [71] assumed that the reduction in sugar contents due to salinity may be associated with the unavailability of carbohydrates, as a consequence of the inhibition in the photosynthesis process. It is worth noting that the inoculation of the endophytic fungus *Aspergillus japonicus* increased the contents of soluble proteins in soybean and sunflower plants as compared to untreated plants either under normal or stress conditions [72]. Moreover, wheat plants subjected to salt stress and colonized by endophytic fungi (*Alternaria chlamydospora*, *Fusarium equiseti*, *Chaetomium coarctatum*, and *Fusarium graminearum*) possess a greater sugar content than uninoculated plants [70]. Previous and recent reports confirmed that *Aspergillus aculeatus* could mitigate the detrimental effects of some environmental stresses, thereby enhancing plant growth via modulating synthesis, degradation, and the storage of sugars to enhance salt resistance [73,74]. Moreover, Robert-Seilaniantz et al. [75] implied that, under salinity stress conditions, plant-associated endophytic fungi can increase the sugar content as an osmo-protectant. High levels of sugars in mycorrhizal plants suggest that they have a role in salinity tolerance [76]. Many reports suggested that fungal endophytes protect the plants from environmental stress by enhancing their antioxidant activity. This change promotes sugar accumulation, which may lead to the scavenging of ROS or prevent oxidative damage to the cells [77].

It has been previously reported that the exposure of wheat plants to different salinity concentrations (60, 120, and 180 mM) led to significant decreases in soluble protein contents [51]. Likewise, different saline stress levels (100, 200, and 300 mM of NaCl) caused marked reductions in soluble protein contents in rice plants [78]. Early studies indicated that salinity stress caused the depression in protein synthesis [74,79,80], which is probably a major reason for the decline in crude protein content. High soluble protein contents have been recorded in different crop plants when treated with endophytic fungi either under normal or salinity stress conditions [51,78]. In suitable conditions, using *Aspergillus flavus* (CHS1) significantly enhanced protein concentrations in soybean plants compared to non-inoculated plants [10]. Furthermore, the total protein contents of sunflower and soybean were significantly increased after being treated with *A. japonicus* compared with the control plants [72]. Moreover, Radhakrishnan et al. [81] showed that the plant-growth-promoting fungus *Penicillium sp.* (NICS01), which is closely related to the genus *Aspergillus* sp., increased the protein contents of sesame plants grown in a salinized soil. They suggested that soil fungi supply inorganic nutrients, which are essential for the biological activities of plants. The present fungal isolate has been able to produce a plant growth regulator (IAA), a naturally occurring plant hormone. This regulator is known for its beneficial effects on plant growth, especially in plants subjected to environmental stress including salinity [82].

The amino acid proline plays a pivotal role in osmo-protection and was substantially increased in salinity-stressed plants [6,74,78,83], which is in accordance with our findings. In a recent study, Bouzouina et al. [70] documented that salinity stress caused a notable aggregation of proline contents in wheat plants. In common bean plants, proline levels were substantially increased as a consequence of salinity exposure [69]. Endophytic fungi could enhance the ability of plants to alleviate salinity stress through coordinating with the amino acids to exert a protective mechanism [74]. The treatment with the endophytic fungus *Aspergillus japonicus* caused significant reductions in the contents of proline in the hosted plants (soybean and sunflower) under unsuitable conditions [72]. Recently, upon the treatment with the endophytic fungus isolate *Piriformospora indica*, proline contents in rice plants were suppressed in salt-stressed plants [84]. The reduction in the proline content in stressed-barley plants as a result of the treatment with the endophytic fungus *A. ochraceus* may be attributed to the fungus’ antioxidant activity, which enables it to scavenge ROS and thus mitigate the osmotic stress. Therefore, proline, as a stress indicator, appeared in a low concentration.

The induction of antioxidant enzymes is an important mechanism to cope with salinity-induced oxidative stress, and our findings in barley plants are in accordance with those in different crops, such as barley [85], common bean [69,86], lettuce [87], wheat plants [88], and maize and rice [50]. In an earlier study, Siddiqui et al. [63] reported that the activities of antioxidant enzymes like SOD and POD were increased when mung bean plants were subjected to different saline levels. The results of Asaf et al. [10] demonstrated that NaCl-induced stress levels (150 mM, 300 mM, and 400 mM) significantly increased the activity of SOD, PPO, and POD in soybean plants. Recently, Jan et al. [52], in their study on maize plants, indicated that salinity stress has also caused a significant increase in the activities of peroxidases. Moreover, salinity stress produces radical species known as ROS, like hydroxyl, superoxide, and hydrogen peroxide, which causes negative effects on the plants’ cell, as ion toxicity, osmotic stress, and oxidative damage. Antioxidant enzymes control these negative effects by reducing the production of free radicals and manage the redox homeostasis in the cell through absorbing oxidants and reducing agents [89]. With respect to the application of the endophytic fungus *A. ochraceus*, our results are in agreement with those reported by Asaf et al. [10], who revealed that the presence of the *Aspergillus flavus* (CHS1) isolate in combination with NaCl induced the activities of SOD, PPO, and POD enzymes in soybean plants, compared to fungus-free plants under NaCl stress. Moreover, they reported that fungal-associated plants tolerated saline conditions by reducing ROS through the activities of antioxidant enzymes. Furthermore, the investigation by Li et al. [74] submitted that the application of *A. aculeatus* could contribute to the detoxification of H_2_O_2_ by promoting peroxidase activities under saline stress, and the antioxidants might be playing a critical role in the *A. aculeatus*-mediated plant tolerance to salinity. It was observed that the applications of endophytic fungi help in the mitigation of salinity stress by elevating the activities of numerous detoxifying enzymes [68,90,91,92].

Abiotic stresses like salinity stress influence the growth and development of plants and stimulate the generation of ROS [6,93]. The generated reactive oxygen species caused severe damage to plant cells through the destruction of cell membranes, which results in an increase of malondialdehyde content. In our study, the reported increase in MDA and H_2_O_2_ contents in barley leaves is due to the fact that many components, including protein and lipids, join the formation of the plant cell membranes, and salt stress breaks these components down within the cell membranes as a result of the increased development of ROS, which also affects other cellular components, such as carbohydrates and protein [6,94,95,96]. Endophytic fungi play a major role in combating abiotic stresses, specifically salinity stress [53,97]. Seed priming with *Aspergillus ochraceus* lessened the contents of MDA and H_2_O_2_ in barley leaves. The magnitude decreases in oxidative stress indicators by the *Aspergillus ochraceus* extract can be attributed to the role of the fungus extract in diminishing salinity stress by acting as a mediator that allows barley to stimulate a stress-responsive system, or through the regulation of the synthesis of metabolites as soluble carbohydrates and soluble protein [73,74].

Salinity is the key abiotic element which decreases seedlings’ growth and yield by inducing hyperosmotic and hyperionic effects on the rhizosphere of the soil [6,93]. The reduction in crop yield may be due to a limitation in growth of stressed barley plants and a reduction of photosynthetic pigments in barley leaves. Our results are in line with those of other investigators [27,98,99,100]. Moreover, the enhancement of barley yield due to the application of *Aspergillus ochraceus* may be attributed to the stimulatory effect of the IAA-producing fungus *Aspergillus ochraceus*, which scavenges ROS and enhances the growth of stressed plants. Several studies indicate the role of endophytic fungi and IAA in diminishing the salinity stress effect on the growth and yield of different plants [74,97,101,102]. The enhancement of the yield of barley plants in response to the application of *Aspergillus ochraceus* is linked with positive changes in the growth characters of barley plants.

## 4. Materials and Methods

### 4.1. Fungal Endophytes and Growth Condition

In our study, seven fungal endophyte strains were previously isolated from leaves of *Avicennia marina* growing in a semi-arid environment. The fungal endophytes *Aspergillus hiratsukae*, *Alternaria tenuissima*, *Chaetomium* sp., *Curvularia lunata*, *Chaetomium* sp., *Chaetomium globosum*, and *Aspergillus ochraceus* were deposited in a gene bank with the accession numbers MT089951, MT089952, MT089953, MT089955, MT089956, MT089957, and MT089958, respectively [45]. All strains were inoculated on malt extract agar (MEA) (Oxoid) plates, incubated for 3–5 days at 28 ± 2 °C, and then kept at 4 °C for further use [103].

### 4.2. Screening of Fungal Endophyte Strains according to Salt Tolerance

To evaluate the ability of fungal endophyte strains to tolerate salt, different concentrations of sodium chloride (50, 100, 150, 200, and 250 g L^−1^) were amended with an MEA medium. Then, the fungal endophyte strains were inoculated on the surface medium at each concentration individually and incubated for 21 days at 30 °C.

### 4.3. Indole Acetic Acid Production

*Aspergillus ochraceus* was tested for IAA production. To do so, *A. ochraceus* was cultured on a malt extract broth (Oxoid) supplemented with tryptophan at different concentrations (1, 2, 5 and 10 g L^−1^) individually and at different seawater levels (0, 15, and 30%). Then, it was incubated for 14 days at 30 ± 2 °C. After the incubation period, a filtration was performed: 1 mL of filtrate was mixed with 4 mL of Salkowski reagent and incubated at room temperature for 20 min. The appearance of a pink color indicates indole formation. The absorbance of the sample was measured at 530 nm, and the quantity of IAA was calculated according to the IAA standard graph [104].

### 4.4. Antioxidant Activity of Crude Extracts of A. ochraceus

*A. ochraceus* was cultured on MEA media containing 0, 15%, and 30% seawater, then incubated for 7 days at 28 ± 2 °C. Crude extracts were assessed for antioxidant activity at different concentrations (1000, 500, 250, 125, 62.5, 31.25, 15.62, and 7.81 µg mL^−1^) according to the DPPH method described by Khalil et al. [45], with minor modifications.

### 4.5. Antifungal Activity

The antifungal activity of crude extracts of *A. ochraceus* in the case of 0, 15%, and 30% seawater was evaluated against phytopathogenic *Fusarium oxysporum* RCMB 08213, which was purchased from the Regional Center of Mycology and Biotechnology, Al-Azhar University, Cairo, Egypt. The antifungal activity was evaluated according to the method used by Khalil et al. [45], with modifications. Additionally, the minimum inhibitory concentration (MIC) for each extract was determined using the broth micro-dilution method [105]. Initially, 100 μL of broth medium was added in a 96-well plate. Fungal suspensions were prepared to be 107 spores/mL and were cultured in a 96-well plate containing a broth medium. Two-fold serial dilutions of crude extracts of *A. ochraceus* were performed to obtain a final concentration range from 1000 to 0.008 µg/L, and 100 μL was added to each dilution. The microtiter plates with 96 U wells were incubated for 3 days at 30 °C.

### 4.6. Pot Experiment

A pot experiment was carried out in the botanical garden of the botany and microbiology department in Cairo, Egypt, during the winter season of 2019. Sixty pots (30 cm in diameter) were filled with sandy-loamy soil (9 kg/pot). Seeds of barley (Hordeum vulgare L. cv. Giza 111) were supplied from the Agriculture research center, Dokki, Giza, Egypt. Uniform seeds were surface-sterilized with 1% sodium hypochlorite and then washed several times with distilled water. Thereafter, the seeds were divided into two equal sets. The first was primed with *Aspergillus ochraceus* for 2 h. and the second was soaked in distilled water. Seeds of each set were planted in thirty pots at the rate of 10 seeds/pot. The growth conditions were: average day/night temperature cycle of 29/19, light 11/13 h, and air humidity between 34% and 55%. After 21 days from sowing, the plants were thinned to five plants per pot and the pots within each set were subdivided into three groups for subsequent irrigation with different levels of seawater, viz 0%, 15%, or 30% (*v*/*v*; seawater/distilled water). Seawater was collected from the Red Sea of Ain Sokhna, Egypt. A fungal treatment was repeated twice by foliar spraying with a range of (300 mL/pot), while fungal-free plants were sprayed with distilled water. Samples were taken from each pot for different growth aspects (shoot and root lengths, fresh and dry weights of shoots and roots, number of leaves per plant), along with the biochemical parameters after 49 days from sowing, while yield samples were taken after 120 days.

#### 4.6.1. Biochemical Parameters

##### Estimation of Photosynthetic Pigments

The content of chlorophyll in fresh leaves of barley plants was determined according to a method defined by Vernon and Seely [106]. In it, one gram of fresh leaves was extracted in 100 mL of acetone (80%). The homogenate was filtered, and then the filtrate was diluted to a total volume of 100 mL using acetone (80%). The absorbance of the extract was measured at 470, 649, and 665 nm.

##### Determination of Osmolyte Contents

According to Umbreit et al. [107], the soluble sugar content of dried barley plant shoots was quantified. One gram of sample was extracted in 5 mL of phenol (2%) and 10 mL of trichloroacetic acid (30%). After filtration, 2 mL of the extract were mixed with 4 mL of an anthrone reagent (2 g anthrone/L of 95% sulfuric acid). The developed blue-green color was measured at 620 nm.

In dried shoots of barley plants, the soluble protein content was measured with the method of Lowry et al. [108]. One gram of sample was homogenized with 5 mL of phenol (2%) and 10 mL of distilled water. After filtration, 1 mL of extract was added to 5 mL of alkaline reagent (50 mL of 2% Na_2_CO_3_ prepared in 0.1 N NaOH and 1 mL of 0.5% CuSO_4_·5H_2_O prepared in 1% KNaC_4_H_4_O_6_·4H_2_O) and mixed thoroughly. Then, 0.5 mL of folin reagent was added (diluted 1:3 *v*/*v*). After 30 min, the developed color was measured at 750 nm.

We used the method employed in Bates et al. [109] to estimate the proline content in dried shoots of barley plants. A half gram of the dried sample was mixed with 10 mL sulfosalicylic acid (3%). The mixture was filtered and 2 mL from it were mixed with 2 mL of acid ninhydrin (warm 1.25 g ninhydrin in 30 mL glacial acetic acid and 20 mL 6 M phosphoric acid) and 2 mL of acetic acid (glacial) for 60 min in a boiling water bath. Then, the reaction was placed in an ice bath, and 4 mL of toluene was added to the mixture. The color was read at 520 nm.

##### Extraction and Determination of Antioxidant Enzymes

The antioxidant enzymes peroxidase (POD), superoxide dismutase (SOD), and polyphenol oxidase (PPO) of barley plants were extracted according to methods described in [110]. The activity of peroxidase (POD), superoxide dismutase (SOD) and polyphenol oxidase (PPO) in the extract was estimated according to methods described in [111], [112], and [113], respectively.

##### Estimation of Malondialdehyde Content

Malondialdehyde content (MDA) was estimated according to the methods used by Heath and Packer [114]. Barley fresh leaf samples were extracted by 5% trichloroacetic acid and centrifugated at 4000 *g* for 10 min. Then, 2 mL of the extract were mixed with 2 mL of a 0.6% Thiobarbituric acid (TBA) solution, and the mixture was placed in a water bath for 10 min. After cooling, the absorbance of the devolved color was at 532, 600, and, subsequently, 450 nm. Malondialdehyde was calculated according to the following equation: 6.45 × (A_532_ − A_600_) − 0.56 × A_450_.

##### Determination of Hydrogen Peroxide (H_2_O_2_) Content

The hydrogen peroxide (H_2_O_2_) content in fresh leaves of barley plants were estimated according to methods described in [110]. Half a gram of fresh leaves was mixed with 4 mL of cold acetone for extraction and then filtrated. Afterwards, 3 mL of solution was mixed with 1% titanium dioxide dissolved in 20% H_2_SO_4_, and then the mixture was centrifugated at 6000× *g* for 15 min. The developed yellow color was then measured at 415 nm.

### 4.7. Statistical Analysis

To assess the significance difference between the treatments, we used CoStat (CoHort software, Monterey, CA, USA). Data based on three replicates (*n* = 3) were subjected to a one-way variance analysis (ANOVA). A least significant difference (LSD) test was applied to compare the mean values at *p* < 0.05.

## 5. Conclusions

In the current study, *A. ochraceus* MT089958 was used for plant growth promotion as well as an antifungal agent at different irrigation levels. Fungal endophyte strains were screened for salt tolerance; the results showed that *A. ochraceus* ranked highest with regard to salt tolerance. In vitro, *A. ochraceus* produces IAA and antioxidant compounds at 15 and 30% seawater, higher than 0% seawater. Moreover, an *A. ochraceus* extract at 15 and 30% seawater exhibited antifungal activity toward *F. oxysporum*. In vivo, the endophytic fungus *A. ochraceus* overcame the deleterious effects of salinity and caused significant increases in the growth traits, leaf pigments, sugars, protein contents, and yield criteria of barley plants grown under salinity stress (either at 15 or 30% seawater). Additionally, using *A. ochraceus* inhibited the amount of proline, malondialdehyde, and hydrogen peroxide, but increased the activities of antioxidant enzymes of barley plants as a mitigation method for the developed damage from salinity stress. Therefore, the current study recommends using the endophytic fungus *A. ochraceus* MT089958 as a way of promoting plant growth in normal and salinity conditions. In addition, this strategy is eco-friendly and easy to apply in the field for regulating and boosting the growth, metabolic activities, and production of different crops.

## Figures and Tables

**Table 1:** Screening of salt-tolerant endophytic fungi.

Fungal Endophyte Name	Accession Number	Fungal Growth at Different Concentrations of NaCl g L^−1^				
		50	100	150	200	250
*Aspergillus hiratsukae*	MT089951	++	+	−	−	−
*Alternaria tenuissima*	MT089952	+++	++	+	−	−
*Chaetomium* sp.	MT089953	++	+	−	−	−
*Curvularia lunata*	MT089955	+++	++	+	−	−
*Chaetomium* sp.	MT089956	++	+	−	−	−
*Chaetomium globosum*	MT089957	++	+	−	−	−
*Aspergillus ochraceus*	MT089958	+++	+++	++	+	−

+++ means high growth, ++ means moderate growth, + means low growth, and − means no growth.

**Table 2:** Antifungal activity of the crude extract of *A. ochraceus* cultured on different salinity levels against *F. oxysporum* at different levels of salinity.

Treatment no.	Inhibition Zone (mm) (1000 µg/mL)	MIC (µg/mL)
0% seawater	0 ^c^	Not Detected
15% seawater	17.3 ± 0.57 ^b^	250
30% seawater	23.6 ± 1.15 ^a^	62.5

Different letters indicate salinity at different levels.

**Table 3:** Effect of seawater irrigation, *A. ochraceus* application, and their interactions on soluble sugars, soluble proteins, and free proline contents (mg g^−1^ DW) in barley plants. Each value represents the mean ± standard error. Different letters indicate significant differences between the treatments according to an LSD test at *p* < 0.05. DW: Dry weight, S.W.: Seawater.

Treatments		Soluble Sugars	Soluble Protein	Free Proline	
Salinity	0% S.W.	93.09 ± 2.96 b	44.89 ± 0.73 b		0.38 ± 0.01 d
	15% S.W.	78.72 ± 0.90 d	30.67 ± 0.29 e		0.88 ± 0.03 b
	30% S.W.	70.65 ± 2.37 e	25.52 ± 0.57 f		1.00 ± 0.02 a
Salinity *+ A. ochraceus*	0% S.W.	104.08 ± 2.01 a	73.55 ± 0.38 a		0.39 ± 0.002 d
	15% S.W.	86.81 ± 2.46 bc	41.34 ± 0.32 c		0.55 ± 0.001 c
	30% S.W.	80.72 ± 3.76 cd	39.00 ± 0.40 d		0.59 ± 0.003 c

**Table 4:** Effect of seawater irrigation, *A. ochraceus* application, and their interactions on yield characteristics of barley plants. Each value represents the mean ± standard error. The letters indicate significant differences between the treatments according to an LSD test at *p* < 0.05.

Treatments		Spike Length (cm)	Spike Weight (g plant^−1^)	Number of Grains	Grains Weight (g plant^−1^)	1000 Grains Weight (g plant^−1^)
**Salinity**	0% S.W.	15.93 ± 0.17 a	2.95 ± 0.25 ab	28.33 ± 1.02 a	2.18 ± 0.15 ab	73.33 ± 1.20 ab
	15% S.W.	12.7 ± 0.24 bc	1.82 ± 0.23 d	16.66 ± 1.20 b	1.34 ± 0.17 c	56.66 ± 1.20 c
	30% S.W.	9.96 ± 0.44 d	1.33 ± 0.23 d	9.33 ± 1.45 c	1.09 ± 0.20 c	34.33 ± 2.40 e
**Salinity + *A. ochraceus***	0% S.W.	16.3 ± 0.62 a	3.64 ± 0.15 a	32.33 ± 1.47 a	2.7 ± 0.04 a	78.66 ± 1.85 a
	15% S.W.	15.16 ± 0.57 ab	2.74 ± 0.28 bc	26.66 ± 1.21 a	2.12 ± 0.21 b	68.33 ± 2.84 b
	30% S.W.	12.5 ± 1.04 c	1.98 ± 0.41 cd	14.33 ± 1.51 bc	1.46 ± 0.06 c	45 ± 1.73 e

## Data Availability

The data presented in this study is contained within the article.

## References

[1] Bybordi A. (2012). Effect of different ratios of nitrate and ammonium on photosynthesis, and fatty acid composition of canola under saline conditions. Int. J. Agric. Crop Sci..

[2] Ewais E.A., Ismail M.A., Amin M.A., Badawy A.A. (2015). Efficiency of salicylic acid and glycine on sugar beet plants grown under heavy metals pollution. Egypt. J. Biotechnol..

[3] De Oliveira A.B., Alencar N.L.M., Gomes-Filho E. (2013). Comparison between the water and salt stress effects on plant growth and development. Responses Org. Water Stress.

[4] Siddiqui Z.S., Khan M.A. (2013). Some physiological attributes of dimorphic seeds of *Halopyrum mucronatum* (L.) Stapf. Pakistan J. Bot..

[5] Munns R. (2005). Genes and salt tolerance: Bringing them together. New Phytol..

[6] Osman M.S., Badawy A.A., Osman A.I., Abdel Latef A.A.H. (2021). Ameliorative impact of an extract of the halophyte Arthrocnemum macrostachyum on growth and biochemical parameters of soybean under salinity stress. J. Plant Growth Regul..

[7] Ren S., Lyle C., Jiang G., Penumala A. (2016). Soybean salt tolerance 1 (GmST1) reduces ROS production, enhances ABA sensitivity, and abiotic stress tolerance in Arabidopsis thaliana. Front. Plant Sci..

[8] Atzori G., de Vos A.C., van Rijsselberghe M., Vignolini P., Rozema J., Mancuso S., van Bodegom P.M. (2017). Effects of increased seawater salinity irrigation on growth and quality of the edible halophyte *Mesembryanthemum crystallinum* L. under field conditions. Agric. Water Manag..

[9] Abdel Latef A.A.H., Mostofa M.G., Rahman M.M., Abdel-Farid I.B., Tran L.P. (2019). Extracts from yeast and carrot roots enhance maize performance under seawater-induced salt stress by altering physio-biochemical characteristics of stressed plants. J. Plant Growth Regul..

[10] Asaf S., Hamayun M., Khan A.L., Waqas M., Khan M.A., Jan R., Lee I.J., Hussain A. (2018). Salt tolerance of Glycine max. L induced by endophytic fungus *Aspergillus flavus* CSH1, via regulating its endogenous hormones and antioxidative system. Plant Physiol. Biochem..

[11] Powthong P., Jantrapanukorn B., Thongmee A., Suntornthiticharoen P. (2013). Screening of antimicrobial activities of the endophytic fungi isolated from *Sesbania grandiflora* (L.) pers. J. Agric. Sci. Technol..

[12] Pavithra G., Bindal S., Rana M., Srivastava S. (2020). Role of endophytic microbes against plant pathogens: A review. Asian J. Plant Sci..

[13] Rana K.L., Kour D., Kaur T., Devi R., Yadav A.N., Yadav N., Dhaliwal H.S., Saxena A.K. (2020). Endophytic microbes: Biodiversity, plant growth-promoting mechanisms and potential applications for agricultural sustainability. Antonie Van Leeuwenhoek.

[14] Abro M.A., Sun X., Li X., Jatoi G.H., Guo L.-D. (2019). Biocontrol potential of fungal endophytes against *Fusarium oxysporum* f. sp. cucumerinum causing wilt in cucumber. Plant Pathol. J..

[15] González V., Armijos E., Garcés-Claver A. (2020). Fungal endophytes as biocontrol agents against the main soil-borne diseases of melon and watermelon in Spain. Agronomy.

[16] Wei F., Zhang Y., Shi Y., Feng H., Zhao L., Feng Z., Zhu H. (2019). Evaluation of the biocontrol potential of endophytic fungus *Fusarium solani* CEF559 against Verticillium dahliae in cotton plant. Biomed. Res. Int..

[17] Gasoni L., de Gurfinkel S. (2009). Biocontrol of Rhizoctonia solani by the endophytic fungus *Cladorrhinum foecundissimum* in cotton plants. Australas. Plant Pathol..

[18] Chowdhary K., Sharma S. (2020). Plant Growth Promotion and Biocontrol Potential of Fungal Endophytes in the Inflorescence of *Aloe vera* L.. Proc. Natl. Acad. Sci. India Sect. B Biol. Sci..

[19] Gurulingappa P., Sword G.A., Murdoch G., McGee P.A. (2010). Colonization of crop plants by fungal entomopathogens and their effects on two insect pests when in planta. Biol. Control..

[20] Salas-Marina M.A., Silva-Flores M.A., Cervantes-Badillo M.G., Rosales-Saavedra M.T., Islas-Osuna M.A., Casas-Flores S. (2011). The plant growth-promoting fungus *Aspergillus ustus* promotes growth and induces resistance against different lifestyle pathogens in *Arabidopsis thaliana*. J. Microbiol Biotechnol..

[21] Yoo S., Shin D.J., Won H.Y., Song J., Kyung M. (2018). *Aspergillus terreus* JF27 promotes the growth of tomato plants and induces resistance against *Pseudomonas syringae* pv. tomato. Mycobiology.

[22] Janardan Y., Verma J.P., Tiwari K.N. (2011). Plant growth promoting activities of fungi and their effect on chickpea plant growth. Asian J. Biol. Sci..

[23] Richardson A.E., Barea J.-M., McNeill A.M., Prigent-Combaret C. (2009). Acquisition of phosphorus and nitrogen in the rhizosphere and plant growth promotion by microorganisms. Plant Soil.

[24] Schulte D., Close T.J., Graner A., Langridge P., Matsumoto T., Muehlbauer G., Sato K., Schulman A.H., Waugh R., Wise R.P. (2009). The international barley sequencing consortium—At the threshold of efficient access to the barley genome. Plant Physiol..

[25] Visioni A., Al-Abdallat A., Elenien J.A., Verma R.P.S., Gyawali S., Baum M. (2019). Genomics and molecular breeding for improving tolerance to abiotic stress in barley (*Hordeum vulgare* L.). Genomics Assisted Breeding of Crops for Abiotic Stress Tolerance, Vol. II..

[26] Mwando E., Angessa T.T., Han Y., Li C. (2020). Salinity tolerance in barley during germination—Homologs and potential genes. J. Zhejiang Univ. B.

[27] Omar M.N.A., Osman M.E.H., Kasim W.A., Abd El-Daim I.A. (2009). Improvement of salt tolerance mechanisms of barley cultivated under salt stress using *Azospirillum brasilense*. Salinity and Water Stress.

[28] Jalili B., Bagheri H., Azadi S., Soltani J. (2020). Identification and salt tolerance evaluation of endophyte fungi isolates from halophyte plants. Int. J. Environ. Sci. Technol..

[29] Morsy M., Cleckler B., Armuelles-Millican H. (2020). Fungal endophytes promote tomato growth and enhance drought and salt tolerance. Plants.

[30] Biango-Daniels M.N., Hodge K.T. (2018). Sea salts as a potential source of food spoilage fungi. Food Microbiol..

[31] González-Martinez S., Soria I., Ayala N., Portillo-López A. (2017). Culturable halotolerant fungal isolates from Southern California Gulf sediments. Open Agric..

[32] Martinelli L., Zalar P., Gunde-Cimerman N., Azua-Bustos A., Sterflinger K., Piñar G. (2017). *Aspergillus atacamensis* and *A. salisburgensis*: Two new halophilic species from hypersaline/arid habitats with a phialosimplex-like morphology. Extremophiles.

[33] Dastogeer K.M.G., Li H., Sivasithamparam K., Wylie S.J. (2018). In vitro salt and thermal tolerance of fungal endophytes of *Nicotiana* spp. growing in arid regions of north-western Australia. Arch. Phytopathol. Plant Prot..

[34] Kunčič M.K., Kogej T., Drobne D., Gunde-Cimerman N. (2010). Morphological response of the halophilic fungal genus Wallemia to high salinity. Appl. Environ. Microbiol..

[35] Ikram M., Ali N., Jan G., Jan F.G., Rahman I.U., Iqbal A., Hamayun M. (2018). IAA producing fungal endophyte *Penicillium roqueforti* Thom., enhances stress tolerance and nutrients uptake in wheat plants grown on heavy metal contaminated soils. PLoS ONE.

[36] Kuswinanti T., Syam’un E., Masniawati A. (2015). The potency of endophytic fungal isolates collected from local aromatic rice as indole acetic acid (IAA) producer. Procedia Food Sci..

[37] Mehmood A., Hussain A., Irshad M., Hamayun M., Iqbal A., Khan N. (2019). In vitro production of IAA by endophytic fungus *Aspergillus awamori* and its growth promoting activities in Zea mays. Symbiosis.

[38] Ahmad F., Ahmad I., KHAN M.S. (2005). Indole acetic acid production by the indigenous isolates of Azotobacter and fluorescent Pseudomonas in the presence and absence of tryptophan. Turk. J. Biol..

[39] Turbat A., Rakk D., Vigneshwari A., Kocsubé S., Thu H., Szepesi Á., Bakacsy L., Škrbić B.D., Jigjiddorj E.-A., Vágvölgyi C. (2020). Characterization of the plant growth-promoting activities of endophytic fungi isolated from sophora flavescens. Microorganisms.

[40] Fouda A.H., Hassan S.E.-D., Eid A.M., Ewais E.E.-D. (2015). Biotechnological applications of fungal endophytes associated with medicinal plant *Asclepias sinaica* (Bioss.). Ann. Agric. Sci..

[41] Hamilton C.E., Gundel P.E., Helander M., Saikkonen K. (2012). Endophytic mediation of reactive oxygen species and antioxidant activity in plants: A review. Fungal Divers..

[42] Cui J.-L., Guo T.-T., Ren Z.-X., Zhang N.-S., Wang M.-L. (2015). Diversity and antioxidant activity of culturable endophytic fungi from alpine plants of *Rhodiola crenulata*, *R. angusta*, and *R. sachalinensis*. PLoS ONE.

[43] Rahmawati S.I., Izzati F.N., Hapsari Y., Septiana E., Rachman F., Simanjuntak P. (2019). Endophytic microbes and antioxidant activities of secondary metabolites from mangroves *Avicennia marina* and *Xylocarpus granatum*. IOP Conf. Ser. Earth Environ. Sci..

[44] Ravindran C., Naveenan T., Varatharajan G.R., Rajasabapathy R., Meena R.M. (2012). Antioxidants in mangrove plants and endophytic fungal associations. Bot. Mar..

[45] Khalil A.M.A., Abdelaziz A.M., Khaleil M.M., Hashem A.H. (2020). Fungal endophytes from leaves of *Avicennia marina* growing in semi-arid environment as a promising source for bioactive compounds. Lett. Appl. Microbiol..

[46] Gupta S., Schillaci M., Walker R., Smith P.M.C., Watt M., Roessner U. (2020). Alleviation of salinity stress in plants by endophytic plant-fungal symbiosis: Current knowledge, perspectives and future directions. Plant Soil.

[47] Zhang F., Wang Y., Liu C., Chen F., Ge H., Tian F., Yang T., Ma K., Zhang Y. (2019). *Trichoderma harzianum* mitigates salt stress in cucumber via multiple responses. Ecotoxicol. Environ. Saf..

[48] Firáková S., Šturdiková M., Múčková M. (2007). Bioactive secondary metabolites produced by microorganisms associated with plants. Biologia.

[49] Rashmi M., Kushveer J.S., Sarma V.V., Jha S. (2018). Secondary metabolites produced by endophytic fungi from marine environments. Endophytes and Secondary Metabolites.

[50] Yasmeen R., Siddiqui Z.S. (2017). Physiological responses of crop plants against *Trichoderma harzianum* in saline environment. Acta Bot. Croat..

[51] Ikram M., Ali N., Jan G., Iqbal A., Hamayun M., Jan F.G., Hussain A., Lee I.-J., Ikram M., Ali N. (2019). *Trichoderma reesei* improved the nutrition status of wheat crop under salt stress. J. Plant Interact..

[52] Jan F.G., Hamayun M., Hussain A., Iqbal A., Jan G., Khan S.A., Khan H., Lee I.-J., Iqbal A., Jan G. (2019). A promising growth promoting *Meyerozyma caribbica* from *Solanum xanthocarpum* alleviated stress in maize plants. Biosci. Rep..

[53] Zhang S., Gan Y., Xu B. (2016). Application of plant-growth-promoting fungi *Trichoderma longibrachiatum* T6 enhances tolerance of wheat to salt stress through improvement of antioxidative defense system and gene expression. Front. Plant Sci..

[54] Chung Y.S., Kim K.-S., Hamayun M., Kim Y. (2019). Silicon confers soybean resistance to salinity stress through regulation of reactive oxygen and reactive nitrogen species. Front. Plant Sci..

[55] Pandya N.D., Desai P.V., Jadhav H.P., Sayyed R.Z. (2018). Plant growth promoting potential of *Aspergillus* sp. NPF7, isolated from wheat rhizosphere in South Gujarat, India. Environ. Sustain..

[56] Harman G.E., Howell C.R., Viterbo A., Chet I., Lorito M. (2004). *Trichoderma* species—Opportunistic, avirulent plant symbionts. Nat. Rev. Microbiol..

[57] Shoresh M., Harman G.E., Mastouri F. (2010). Induced systemic resistance and plant responses to fungal biocontrol agents. Annu. Rev. Phytopathol..

[58] Khan A.L., Hamayun M., Ahmad N., Hussain J., Kang S.-M., Kim Y.-H., Adnan M., Tang D.-S., Waqas M., Radhakrishnan R. (2011). Salinity stress resistance offered by endophytic fungal interaction between *Penicillium minioluteum* LHL09 and Glycine max. J. Microbiol. Biotechnol..

[59] Estrada B., Aroca R., Barea J.M., Ruiz-Lozano J.M. (2013). Native arbuscular mycorrhizal fungi isolated from a saline habitat improved maize antioxidant systems and plant tolerance to salinity. Plant Sci..

[60] Sairam R.K., Rao K.V., Srivastava G.C. (2002). Differential response of wheat genotypes to long term salinity stress in relation to oxidative stress, antioxidant activity and osmolyte concentration. Plant Sci..

[61] Parida A.K., Das A.B. (2005). Salt tolerance and salinity effects on plants: A review. Ecotoxicol. Environ. Saf..

[62] Aftab T., Khan M.M.A., da Silva J.A.T., Idrees M., Naeem M. (2011). Role of salicylic acid in promoting salt stress tolerance and enhanced artemisinin production in *Artemisia annua* L.. J. Plant Growth Regul..

[63] Siddiqui Z., Cho J.-I., Park S.-H., Kwon T.-R., Lee G.-S., Jeong M.-J., Kim K.-W., Lee S.-K., Park S.-C. (2014). Phenotyping of rice in salt stress environment using high-throughput infrared imaging. Acta Bot. Croat..

[64] Mishra A., Salokhe V.M. (2011). Rice root growth and physiological responses to SRI water management and implications for crop productivity. Paddy Water Environ..

[65] Vafadar F., Amooaghaie R., Otroshy M. (2014). Effects of plant-growth-promoting rhizobacteria and arbuscular mycorrhizal fungus on plant growth, stevioside, NPK, and chlorophyll content of *Stevia rebaudiana*. J. Plant Interact..

[66] Rawat L., Singh Y., Shukla N., Kumar J. (2011). Alleviation of the adverse effects of salinity stress in wheat (*Triticum aestivum* L.) by seed biopriming with salinity tolerant isolates of *Trichoderma harzianum*. Plant Soil.

[67] Zhang F., Yuan J., Yang X., Cui Y., Chen L., Ran W., Shen Q. (2013). Putative *Trichoderma harzianum* mutant promotes cucumber growth by enhanced production of indole acetic acid and plant colonization. Plant Soil.

[68] Hashem A., Abd_Allah E.F., Alqarawi A.A., Al Huqail A.A., Egamberdieva D. (2014). Alleviation of abiotic salt stress in *Ochradenus baccatus* (Del.) by *Trichoderma hamatum* (Bonord.) Bainier. J. Plant Interact..

[69] Younesi O., Moradi A. (2014). Effects of plant growth-promoting rhizobacterium (PGPR) and arbuscular mycorrhizal fungus (AMF) on antioxidant enzyme activities in salt-stressed bean (*Phaseolus vulgaris* L.). Agriculture.

[70] Bouzouina M., Kouadria R., Lotmani B. (2020). Fungal endophytes alleviate salt stress in wheat in terms of growth, ion homeostasis and osmoregulation. J. Appl. Microbiol..

[71] Goicoechea N., Merino S., Sánchez-Díaz M. (2005). Arbuscular mycorrhizal fungi can contribute to maintain antioxidant and carbon metabolism in nodules of *Anthyllis cytisoides* L. subjected to drought. J. Plant Physiol..

[72] Hamayun M., Hussain A., Iqbal A., Khan S.A., Lee I.-J. (2018). Endophytic fungus *Aspergillus japonicus* mediates host plant growth under normal and heat stress conditions. Biomed. Res. Int..

[73] Xie Y., Luo H., Du Z., Hu L., Fu J. (2014). Identification of cadmium-resistant fungi related to Cd transportation in bermudagrass [*Cynodon dactylon* (L.) Pers.]. Chemosphere.

[74] Li X., Han S., Wang G., Liu X., Amombo E. (2017). The fungus aspergillus aculeatus enhances salt-stress tolerance, metabolite accumulation, and improves forage quality in perennial ryegrass. Front. Microbiol..

[75] Robert-Seilaniantz A., Navarro L., Bari R., Jones J.D.G. (2007). Pathological hormone imbalances. Curr. Opin. Plant Biol..

[76] Manchanda G., Garg N. (2011). Alleviation of salt-induced ionic, osmotic and oxidative stresses in Cajanus cajan nodules by AM inoculation. Plant Biosyst..

[77] Gundel P.E., Martínez-Ghersa M.A., Omacini M., Cuyeu R., Pagano E., Ríos R., Ghersa C.M. (2012). Mutualism effectiveness and vertical transmission of symbiotic fungal endophytes in response to host genetic background. Evol. Appl..

[78] Bagheri A.A., Saadatmand S., Niknam V., Nejadsatari T., Babaeizad V. (2013). Effect of endophytic fungus, Piriformospora indica, on growth and activity of antioxidant enzymes of rice (*Oryza sativa* L.) under salinity stress. Int. J. Adv. Biol. Biomed. Res..

[79] Pessarakli M., Huber J.T. (1991). Biomass production and protein synthesis by alfalfa under salt stress. J. Plant Nutr..

[80] Mohamed H.I., Gomaa E.Z. (2012). Effect of plant growth promoting *Bacillus subtilis* and *Pseudomonas fluorescens* on growth and pigment composition of radish plants (*Raphanus sativus*) under NaCl stress. Photosynthetica.

[81] Radhakrishnan R., Kang S., Baek I., Lee I. (2014). Characterization of plant growth-promoting traits of *Penicillium* species against the effects of high soil salinity and root disease. J. Plant Interact..

[82] Devi R.G., Pandiyarajan V., Gurusaravanan P. (2012). Alleviating effect of IAA on salt stressed *Phaseolus mungo* (L.) with reference to growth and biochemical characteristics. Recent Res. Sci. Technol..

[83] Bertrand A., Bipfubusa M., Dhont C., Chalifour F.-P., Drouin P., Beauchamp C.J. (2016). Rhizobial strains exert a major effect on the amino acid composition of alfalfa nodules under NaCl stress. Plant Physiol. Biochem..

[84] Sampangi-Ramaiah M.H., Dey P., Jambagi S., Kumari M.M.V., Oelmüller R., Nataraja K.N., Ravishankar K.V., Ravikanth G., Shaanker R.U. (2020). An endophyte from salt-adapted Pokkali rice confers salt-tolerance to a salt-sensitive rice variety and targets a unique pattern of genes in its new host. Sci. Rep..

[85] Baltruschat H., Fodor J., Harrach B.D., Niemczyk E., Barna B., Gullner G., Janeczko A., Kogel K.K., Schäfer P., Schwarczinger I. (2008). Salt tolerance of barley induced by the root endophyte Piriformospora indica is associated with a strong increase in antioxidants. New Phytol..

[86] Dardanelli M.S., de Cordoba F.J.F., Espuny M.R., Carvajal M.A.R., Díaz M.E.S., Serrano A.M.G., Okon Y., Megías M. (2008). Effect of *Azospirillum brasilense* coinoculated with Rhizobium on *Phaseolus vulgaris* flavonoids and Nod factor production under salt stress. Soil Biol. Biochem..

[87] Kohler J., Hernández J.A., Caravaca F., Roldán A. (2009). Induction of antioxidant enzymes is involved in the greater effectiveness of a PGPR versus AM fungi with respect to increasing the tolerance of lettuce to severe salt stress. Environ. Exp. Bot..

[88] Singh R.P., Jha P.N. (2016). The multifarious PGPR Serratia marcescens CDP-13 augments induced systemic resistance and enhanced salinity tolerance of wheat (*Triticum aestivum* L.). PLoS ONE.

[89] Ahammed G.J., Xu W., Liu A., Chen S. (2019). Endogenous melatonin deficiency aggravates high temperature-induced oxidative stress in *Solanum lycopersicum* L.. Environ. Exp. Bot..

[90] Waller F., Achatz B., Baltruschat H., Fodor J., Becker K., Fischer M., Heier T., Hückelhoven R., Neumann C., Von Wettstein D. (2005). The endophytic fungus Piriformospora indica reprograms barley to salt-stress tolerance, disease resistance, and higher yield. Proc. Natl. Acad. Sci. USA.

[91] Jogawat A., Saha S., Bakshi M., Dayaman V., Kumar M., Dua M., Varma A., Oelmüller R., Tuteja N., Johri A.K. (2013). *Piriformospora indica* rescues growth diminution of rice seedlings during high salt stress. Plant Signal. Behav..

[92] Ahmad P., Hashem A., Abd-Allah E.F., Alqarawi A.A., John R., Egamberdieva D., Gucel S. (2015). Role of *Trichoderma harzianum* in mitigating NaCl stress in Indian mustard (*Brassica juncea* L) through antioxidative defense system. Front. Plant Sci..

[93] Abdel Latef A.A.H., Omer A.M., Badawy A.A., Osman M.S., Ragaey M.M. (2021). Strategy of salt tolerance and interactive impact of *Azotobacter chroococcum* and/or *Alcaligenes faecalis* inoculation on canola (*Brassica napus* L.) plants grown in saline soil. Plants.

[94] Darvishan M., Tohidi-Moghadam H.R., Zahedi H. (2013). The effects of foliar application of ascorbic acid (vitamin C) on physiological and biochemical changes of corn (*Zea mays* L) under irrigation withholding in different growth stages. Maydica.

[95] Yousuf P.Y., Ahmad A., Hemant Ganie A.H., Aref I.M., Iqbal M. (2015). Potassium and calcium application ameliorates growth and oxidative homeostasis in salt-stressed Indian mustard (*Brassica juncea*) plants. Pak. J. Bot.

[96] Nessim A., Kasim W. (2019). Physiological impact of seed priming with CaCl2 or carrot root extract on lupinus termis plants fully grown under salinity stress. Egypt. J. Bot..

[97] Ripa F.A., Cao W., Tong S., Sun J. (2019). Assessment of plant growth promoting and abiotic stress tolerance properties of wheat endophytic fungi. Biomed. Res. Int..

[98] Zhang Z., Mao B., Li H., Zhou W., Takeuchi Y., Yoneyama K. (2005). Effect of salinity on physiological characteristics, yield and quality of microtubers in vitro in potato. Acta Physiol. Plant.

[99] Stavridou E., Hastings A., Webster R.J., Robson P.R.H. (2017). The impact of soil salinity on the yield, composition and physiology of the bioenergy grass *Miscanthus × giganteus*. Gcb Bioenergy.

[100] Zein F.I., Gaiza E.A., EL-Sanafawy H.M., Talha N.I. (2020). Effect of specific ions, salinity and alkalinity on yield and quality of some Egyptian cotton genotypes. Egypt. J. Soil Sci..

[101] Mohamed A.S., Mahmoud M.G., Sharaf A.E.-M.M. (2016). Interactive effect of Trichoderma viride on broad bean (cv. *Vicia faba* L.) genotypes grown under different salinity stress conditions. Int. J. Ecotoxicol. Ecobiol..

[102] Khalid A., Aftab F. (2020). Effect of exogenous application of IAA and GA 3 on growth, protein content, and antioxidant enzymes of *Solanum tuberosum* L. grown in vitro under salt stress. Cell. Dev. Biol..

[103] Hashem A.H., Hasanin M.S., Khalil A.M.A., Suleiman W.B. (2019). Eco-green conversion of watermelon peels to single cell oils using a unique oleaginous fungus: Lichtheimia corymbifera AH13. Waste Biomass Valorization.

[104] Mohite B. (2013). Isolation and characterization of indole acetic acid ( IAA ) producing bacteria from rhizospheric soil and its effect on plant growth. J. Soil Sci. Plant Nutr..

[105] De Sousa E.S.O., Cortez A.C.A., de Melhem M.S.C., Frickmann H., de Souza J.V.B. (2020). Factors influencing susceptibility testing of antifungal drugs: A critical review of document M27-A4 from the Clinical and Laboratory Standards Institute (CLSI). Braz. J. Microbiol..

[106] Vernon L.P., Seely G.R. (1966). The Chlorophylls.

[107] Umbreit W.W., Burris R.H., Stauffer J.F. (1964). Manometric Techniques: A Manual Describing Methods Applicable to the Study of Tissue Metabolism.

[108] Lowry O.H., Rosebrough N.J., Farr A.L., Randall R.J. (1951). Protein measurement with the folin phenol reagent J Biol Chem 193: 265–275. Find. this Artic. Online.

[109] Bates L.S., Waldren R.P., Teare I.D. (1973). Rapid determination of free proline for water-stress studies. Plant Soil.

[110] Mukherjee S.P., Choudhuri M.A. (1983). Implications of water stress-induced changes in the levels of endogenous ascorbic acid and hydrogen peroxide in Vigna seedlings. Physiol. Plant.

[111] Bergmeyer H.U. (1974). Methods of Enzymatic Analysis.

[112] Marklund S., Marklund G. (1974). Involvement of the superoxide anion radical in the autoxidation of pyrogallol and a convenient assay for superoxide dismutase. Eur. J. Biochem..

[113] Matta A., Dimond A.E. (1963). Symptoms of Fusarium wilt in relation to quantity of fungus and enzyme activity in tomato stems. Phytopathology.

[114] Heath R.L., Packer L. (1968). Photoperoxidation in isolated chloroplasts: I. Kinetics and stoichiometry of fatty acid peroxidation. Arch. Biochem. Biophys..

